# Protein Kinase Cδ Stimulates Proteasome-Dependent Degradation of C/EBPα during Apoptosis Induction of Leukemic Cells

**DOI:** 10.1371/journal.pone.0006552

**Published:** 2009-08-07

**Authors:** Meng Zhao, Xu-Fang Duan, Xu-Yun Zhao, Bo Zhang, Ying Lu, Wei Liu, Jin-Ke Cheng, Guo-Qiang Chen

**Affiliations:** 1 Institute of Health Sciences, Shanghai Institutes for Biological Sciences (SIBS) of Chinese Academy of Sciences and Shanghai Jiao Tong University School of Medicine (SJTU-SM), Shanghai, China; 2 Department of Pathophysiology, Key Laboratory of Cell Differentiation and Apoptosis of Chinese Ministry of Education, Shanghai Jiao Tong University School of Medicine (SJTU-SM), Shanghai , China; 3 The National Laboratory for Oncogenes and Related Genes, Shanghai Jiao Tong University School of Medicine (SJTU-SM), Shanghai, China; Dresden University of Technology, Germany

## Abstract

**Background:**

The precise regulation and maintenance of balance between cell proliferation, differentiation and death in metazoan are critical for tissue homeostasis. CCAAT/enhancer-binding protein alpha (C/EBPα) has been implicated as a key regulator of differentiation and proliferation in various cell types. Here we investigated the potential dynamic change and role of C/EBPα protein during apoptosis induction.

**Methodology/Principal Findings:**

Upon onset of apoptosis induced by various kinds of inducers such as NSC606985, etoposide and others, C/EBPα expression presented a profound down-regulation in leukemic cell lines and primary cells via induction of protein degradation and inhibition of transcription, as assessed respectively by cycloheximide inhibition test, real-time quantitative RT-PCR and luciferase reporter assay. Applying chemical inhibition, forced expression of dominant negative mutant and catalytic fragment (CF) of protein kinase Cdelta (PKCδ), which was proteolytically activated during apoptosis induction tested, we showed that the active PKCδ protein contributed to the increased degradation of C/EBPα protein. Three specific proteasome inhibitors antagonized C/EBPα degradation during apoptosis induction. More importantly, ectopic expression of PKCδ-CF stimulated the ubiquitination of C/EBPα protein, while the chemical inhibition of PKCδ action significantly inhibited the enhanced ubiquitination of C/EBPα protein under NSC606985 treatment. Additionally, silencing of C/EBPα expression by small interfering RNAs enhanced, while inducible expression of C/EBPα inhibited NSC606985/etoposide-induced apoptosis in leukemic cells.

**Conclusions/Significance:**

These observations indicate that the activation of PKCδ upon apoptosis results in the increased proteasome-dependent degradation of C/EBPα, which partially contributes to PKCδ-mediated apoptosis.

## Introduction

The precise regulation and maintenance of balance between cell proliferation, differentiation and death in multicellular organisms are critical for tissue homeostasis. Their disorders would cause the pathogenesis of many diseases, especially cancers including leukemias. Increasing lines of evidence support that the same protein can exert some roles in all or some of these important cellular events. For example, several caspases involved in cell apoptosis were shown to play a role in the differentiation of erythroid cells and macrophages [1]. Anti-apoptotic Bcl-2 is also associated with stem cells-committed differentiation and morphogenesis [2].

CCAAT/enhancer-binding protein alpha (C/EBPα), of which the genetic nomenclature is dubbed CEBPA, has been implicated as a key regulator of differentiation in various cell types such as adipocytes, hepatocytes and myeloid cells [3]–[5]. Its mutations were also found in about 10% of acute myeloid leukemia (AML) [6], and inhibition of its expression or function is also blocked by leukemogenesis-related genetic alterations such as t(8;21)-generated AML1-ETO [7]–[9] or t(9;22)-generated BCR-ABL fusion protein [10]. More recently, C/EBPα was also reported to induce the expression of anti-apoptotic Bcl-2 gene in hematopoietic cell lines in a manner independent of its DNA binding activity and protects Ba/F3 (a widely studied immature murine hematopoietic cell line with a pro-B lymphoid phenotype) from apoptosis on interleukin-3 withdrawal [11]. In this work, we investigate whether C/EBPα expression is modulated during apoptosis and whether C/EBPα contributes to apoptosis development in a common sense. Our observations indicate that the activation of protein kinase Cdelta (PKCδ) upon apoptosis results in the increased proteasome-dependent degradation of C/EBPα, which partially contributes to PKCδ-mediated apoptosis.

## Results

### C/EBPα protein is significantly reduced upon apoptosis induction in leukemic cells

**Figure 1:**
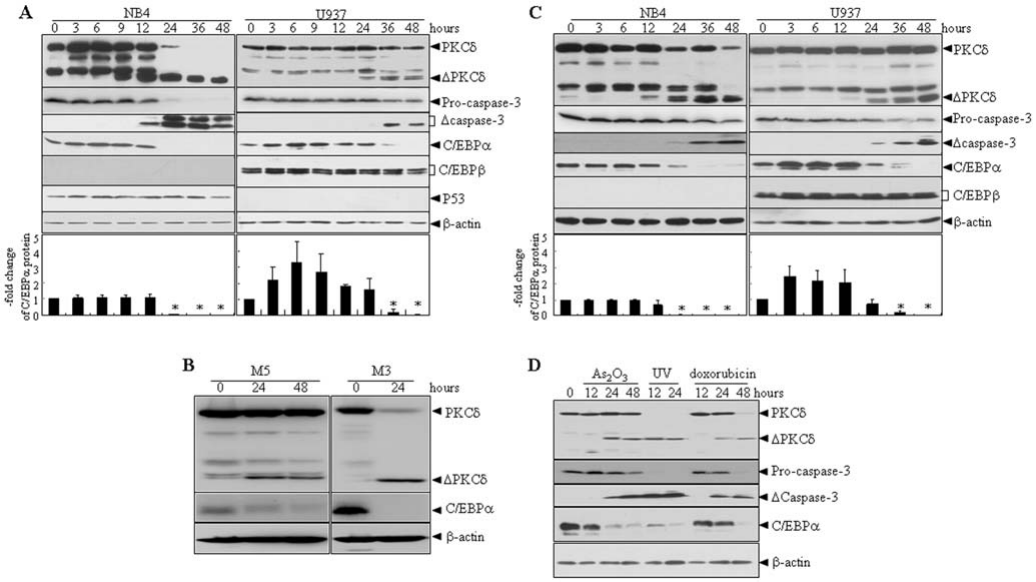
C/EBPα protein is significantly reduced upon the onset of apoptosis in leukemic cells. NB4 and U937 cells were treated respectively with 25 nM and 50 nM of NSC606985 (A) or 0.5 µM and 1 µM etoposide (C). Fresh leukemic cells were treated with 50 nM NSC606985 (B), or NB4 cells were treated with 2 µM As_2_O_3_, 0.5 µM doxorubicin or irradiated by 150J UV for hours as indicated (D). The indicated proteins were detected by Western blots with β-actin as an internal control. Δcaspase-3 and ΔPKCδ indicate activated fragments of caspase-3 and cleaved 41kDa catalytic fragment of PKCδ, respectively. Each experiment was repeated three times with similar results. Fold changes (means±SD from three independent tests) for C/EBPα/β-actin against untreated cells are shown on the bottom of panels A and B. Symbol * represents P value of less than 0.01 compared with the untreated (0 hour) cells.

To evaluate whether the C/EBPα protein is regulated during apoptosis induction, NSC606985 (a water-soluble camptothecin ester derivative [12], [13]) was used as an apoptosis-inducing agent. Consistent with our previous report [14], the proteolytic activation of caspase-3 (a critical apoptosis effector [15]), as assessed by decreased pro-caspase-3 and increased active fragments of caspase-3, was induced by 25 nM of NSC606985 treatment for 12 hours in acute promyelocytic leukemic (APL) NB4 cells or by 50 nM of NSC606985 treatment for 36 hours in acute monocytic leukemic U937 cells (Figure 1A). Concomitantly, the treatment also triggered these two cell lines to undergo apoptosis, in which NB4 cells were more sensitive than U937 cells, as estimated by percentages of annexin-V^+^ cells and sub-G_1_ cells on flow cytometry as well as the Wright's staining-based morphological examinations (Figure S1). When the activated caspase-3 was detected, more intriguingly, C/EBPα protein was significantly reduced to an undetectable level in NSC606985-treated NB4 and U937 cells (Figure 1A). Of note, only the p42 isoform of C/EBPα could be clearly detected in NB4 and U937 cells, although there are two C/EBPα isoforms (p42 and p30) [16]. Therefore, C/EBPα protein is its p42 isoform in the following experiments. Additionally, 50 nM of NSC606985 also effectively reduced C/EBPα protein together with the cleaved activation of PKCδ (Figure 1B) and apoptosis induction (data not shown) in primary leukemic cells from bone marrow (BM) of two AML patients. To determine whether the reduced C/EBPα protein is NSC606985-specific or apoptosis-dependent, other apoptosis inducers including etoposide, doxorubicin, arsenic trioxide (As_2_O_3_) and ultraviolent (UV) radiation were applied to NB4 and U937 cells. All these insults could effectively induce apoptosis of these two cell lines (Figure S2/S3) together with proteolytic activation of caspase-3 (Figure 1C/D). Meanwhile, C/EBPα protein was also reduced in NB4 and U937 cells (Figure 1C/D). Notably, C/EBPβ and p53 proteins were undetectable in NB4 and U937 cells, respectively, and both NSC606985 and etoposide failed to alter the expression of C/EBPβ protein in U937 cells or p53 protein in NB4 cells (Figure 1A/C). All these data supported that the reduction of C/EBPα protein is common to apoptosis induction.

### Both inhibition of transcription and induction of protein degradation contribute to the down-regulation of C/EBPα expression during apoptosis

**Figure 2:**
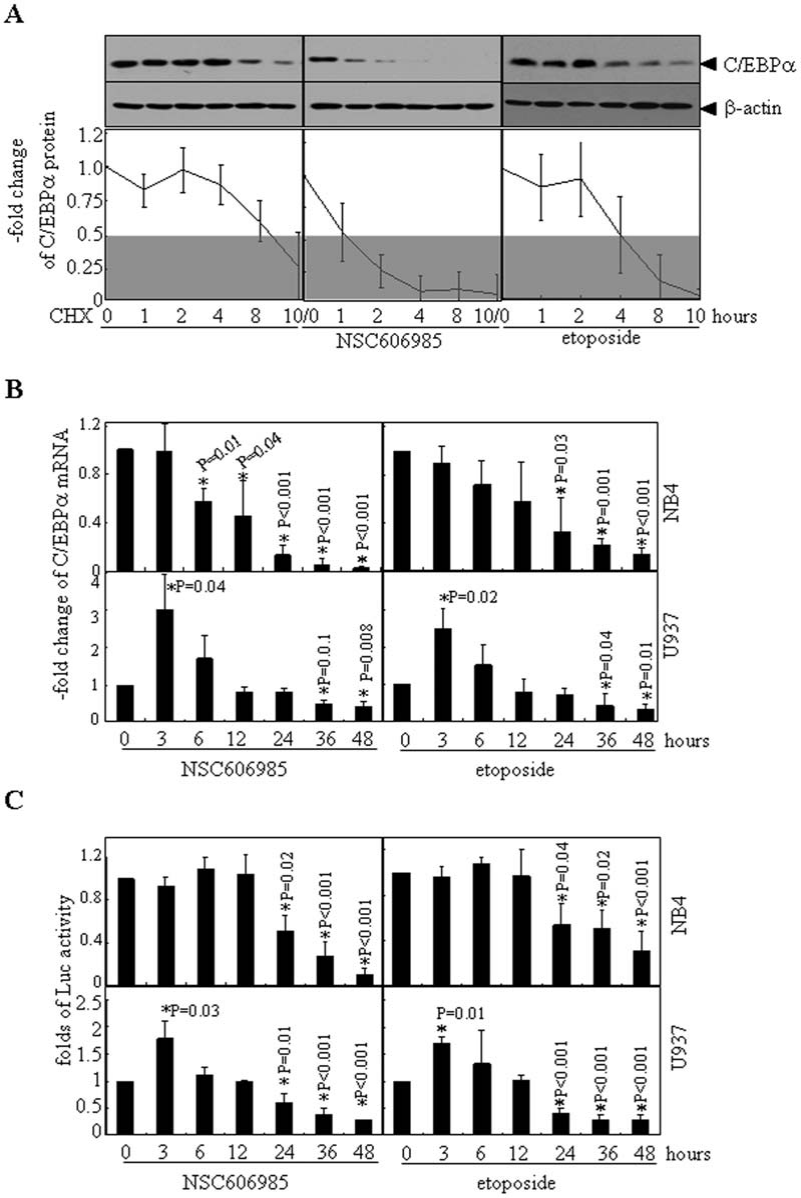
The decrease of C/EBPα protein during apoptosis involves induction of protein degradation and inhibition of transcription. (A) After pretreatment with 25 nM of NSC606985 or 0.5 µM of etoposide for 12 hours, NB4 cells were incubated with 10 µg/ml CHX for indicated hours. Then, C/EBPα protein was detected with β-actin as a loading control. Folds of decrease of C/EBPα protein/β-actin ratios against untreated cells are shown as means±SD of three independent experiments. (B) NB4 (top) and U937 cells(bottom) were treated respectively with 25 nM and 50 nM of NSC606985 (left) or 0.5 µM and 1 µM etoposide (right) for hours as indicated, and CEBPA mRNA level was detected by real-time quantitative RT-PCR. (C) NB4 (top) and U937 cells (bottom) were transfected with CEBPA promotor-luciferase plasmid together with pRL-SV40 vector. After 24 hours of transfection, these cells were treated respectively with 25 nM and 50 nM of NSC606985 (left) or 0.5 µM and 1 µM etoposide (right) for hours as indicated. Then, the cells were harvested and the relative luciferase activities were measured. The columns represent means of fold changes against untreated cells, with the bar as S.D. of three independent experiments each with triplicates The symbol * indicates P value compared with untreated cells.

To test whether C/EBPα expression is regulated at the post-transcriptional level during apoptosis, NB4 cells were treated with 10 µg/ml cycloheximide (CHX) and/or NSC606985 or etoposide for the different times. As shown in Figure 2A, half-life of C/EBPα protein in NB4 cells was more than 8 hours. In NSC606985 or etoposide-treated cells, however, C/EBPα protein disappeared or decreased to less than 50% at 4 hours after CHX blockage of protein synthesis, indicating that the stabilization of the C/EBPα protein was also lowered upon apoptosis induction. Evan so, we continued to dynamically measure the mRNA level of CEBPA in NSC606985- or etoposide-treated NB4 and U937 cells by real-time quantitative RT-PCR. The results revealed that NSC606985/etoposide-treated NB4 cells also presented reduced CEBPA mRNA, which initially appeared at 6 hours and 24 hours after treatment of NSC606985 and etoposide respectively, and became more significant later (Figure 2B). The reduced CEBPA mRNA could also be seen in NSC606985/etoposide-treated U937 cells, but CEBPA mRNA also experienced a rapid but temporary increase before the down-regulation in U937 cells (Figure 2B). This was also true for C/EBPα protein in U937 but not in NB4 cells treated by both NSC606985 and etoposide, although statistic significance was absent (Figure 1A/B). Furthermore, a CEBPA promoter-driven luciferase reporter assay also supported NSC606985 and etoposide significantly inhibited CEBPA gene promoter-driven luciferase transcription in both NB4 and U937 cells (Figure 2C). It should be point out that although the transitory elevation of CEBPA gene promoter-driven transcription could also be tested in U937 cells under the treatment of NSC606985 or etoposide, the early elevation of the reporter gene is very weak which indicated that there may be other mechanisms involved in the transient elevation of CEBPA mRNA. All these results proposed that both reduced transcription and increased degradation contributed to the down-regulation of C/EBPα expression during apoptosis induction.

### The proteolytically activated PKCδ is critical for the enhanced protein degradation of C/EBPα during apoptosis

**Figure 3:**
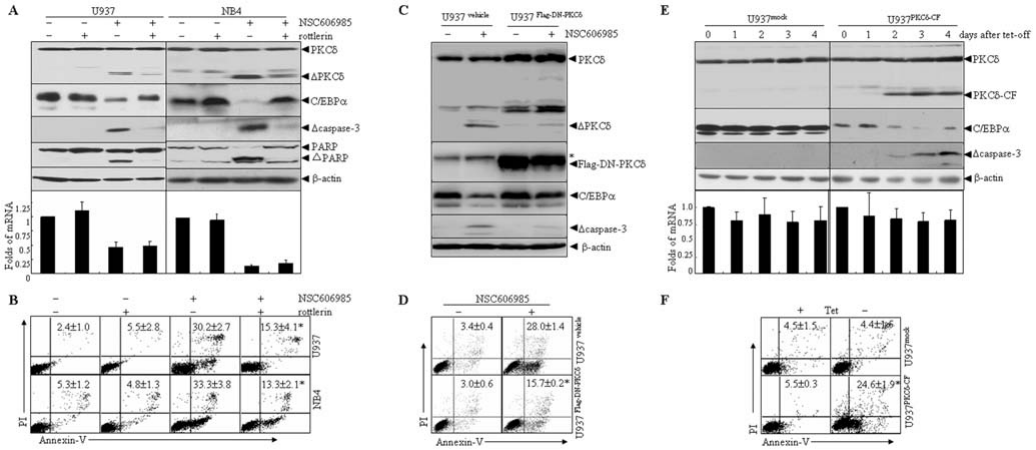
The proteolytically activated PKCδ is critical for the enhanced C/EBPα degradation during apoptosis. (A-B) After pretreatment for 2 hours in the presence or absence of 2 µM and 1 µM of rottlerin respectively for U937 and NB4 cells, U937 cells were treated with or without 50 nM of NSC606985 for an additional 36 hours or NB4 cells were treated with or without 25 nM of NSC606985 for an additional 18 hours. Then, the indicated proteins (top panel, A), relative CEBPA mRNA (bottom panel, A), and annexin-V^+^ cells% (B) were tested. (C-D) U937^Flag-DN-PKCδ^ and U937^vehicle^ cells were treated with 50 nM NSC606985 for 36 hours. Then, the indicated proteins (C) and annexin-V ^+^ cells%(D) were measured. (E) U937^mock^ and U937^PKCδ-CF^ cells were incubated for the indicated days after tetracycline withdrawal, and the indicated proteins (top panel) and relative CEBPA mRNA (bottom panel) were determined. (F) U937^mock^ and U937 ^PKCδ -CF^ cells were incubated in medium with (+) or without (−) tetracycline for 4 days, and annexin-V ^+^ cells% were measured on flow cytometry. Here, Δcaspase-3, ΔPKCδ and ΔPARP indicate activated fragments of caspase-3, cleaved 41kDa catalytic fragments of PKCδ and cleaved fragments of PARP, respectively. In panel C, Flag-DN-PKCδ was detected by anti-Flag antibody, and the symbol * indicated a non-corresponding band. In panel A/E, the columns represent means of change folds of CEBPA mRNA against untreated cells, with the bar as S.D. of three independent experiments with triplicates each. In panel B, D and F, the values represent annexin V^+^ cells with and without PI staining, as expressed by mean±S.D. of three independent experiments each with triplicates, and the symbol * represents P<0.01 compared with NSC606985 treatment alone (B), U937^vehicle^ cells (D) or U937^mock^ cells with the corresponding treatment.

It has been known that PKCδ, a novel member of the PKC family, is implicated as an important regulator of apoptotic responses, especially in DNA-damaging agents-induced apoptosis [14], [17], [18]. The proteolytic activation of PKCδ, which results in the generation of an active kinase domain [19], occurs in response to a variety of stimuli including DNA-damaging agents and exerts a crucial role in NSC606985/etoposide-induced apoptosis [9], [14], [20]. In line with our previous reports [14], [21], NSC606985/etoposide induced a proteolytic cleavage of PKCδ into a 41kDa catalytic fragment (Figure 1A-C) that persistently activated the kinase [20]. Of note, the PKCδ antibody also detected a strong band between the full-length PKCδ and the catalytic fragment (CF) of PKCδ, which also disappeared when cells are treated with apoptotic stimuli. What this band actually corresponds to remained to be explored. Also, it should be pointed out that the increase of truncated PKCδ had a good correlation with the decrease of full-length PKCδ after 24 hours of NSC606985 treatment in NB4 cells, but such correlation was not so remarkable in NSC606985 or etoposide-treated U937 cells. We extrapolated that this might be due to higher sensitivity of NB4 cells than U937 cells to etoposide and especially NSC606985-induced apoptosis (Figure S1/S2). The proteolytic activation of PKCδ was also present in UV, doxorubicin and As_2_O_3_-induced apoptosis (Figure 1D). Considering that the proteolytic activated PKCδ protein could target some proteins for their degradation [22] and the PKCδ activation also occurred before the reduced C/EBPα protein according to time-course analysis (Figure 1A/C and ref [14]), we extrapolated that the PKCδ activation was related to the degradation of C/EBPα protein. Hence, U937 and NB4 cells were pretreated with the specific PKCδ inhibitor rottlerin, which significantly inhibited NSC606985-induced proteolytic activation of PKCδ (Figure 3A). Likewise, it also antagonized activation of caspase-3, cleavage of its substrate poly-ADP ribose polymerase (PARP) (Figure 3A) and apoptosis (Figure 3B) induced by NSC606985. In parallel, rottlerin also partially restored C/EBPα protein but not its mRNA level upon NSC606985 treatment in both cells (Figure 3A).

Since rottlerin directly uncouples mitochondrial respiration from oxidative phosphorylation, and could block any number of ATP-dependent processes or inhibit other kinases [23], [24], data using rottlerin should be evaluated cautiously. Therefore, we also tested the effect of a dominant-negative mutant of PKCδ (DN-PKCδ), which selectively inhibits action of PKCδ [25] and blocks apoptosis in response to diverse apoptotic stimuli [26]. By a retrovirus-infecting system, U937 cell line with stable transfection of the Flag-tagged DN-PKCδ (U937^Flag-DN-PKCδ^) or its control vector (U937^vehicle^) was generated. Our results showed that the forced expression of Flag-DN-PKCδ significantly antagonized NSC606985–induced the activations of caspase-3 and PKCδ (Figure 3C) and apoptosis induction (Figure 3D) in U937^Flag-DN-PKCδ^ cells. Accordingly, DN-PKCδ expression also antagonized the decrease of C/EBPα protein (Figure 3C) but not its mRNA level (Figure S4).

Furthermore, pTRE2hyg-PKCδ-CF-expressing vector and empty vector pTRE2hyg were respectively stably transfected to U937T cells, which contain stably transfected pUHD-tTA (*t*etracycline responsive *t*ranscription *a*ctivator), whose expression is under the control of tetracycline [27]. Here, these transfected cells were referred as the U937^PKCδ-CF^ and U937^mock^ cells, respectively. In U937^PKCδ-CF^ cells but not U937^mock^ cells, PKCδ-CF protein was induced significantly with no alteration of endogenous full-length PKCδ protein from day 2 after tetracycline removal (Figure 3E). In this case, caspase-3 was also activated (Figure 3E) with apoptosis induction (Figure 3F). More intriguingly, the expression of PKCδ-CF also reduced C/EBPα protein but not its mRNA in U937^PKCδ-CF^ cells. All these observations strongly suggested that proteolytic activated PKCδ contributed to the enhanced degradation of C/EBPα protein during apoptosis.

### Activated caspase-3 fails to induce the degradation of C/EBPα protein

**Figure 4:**
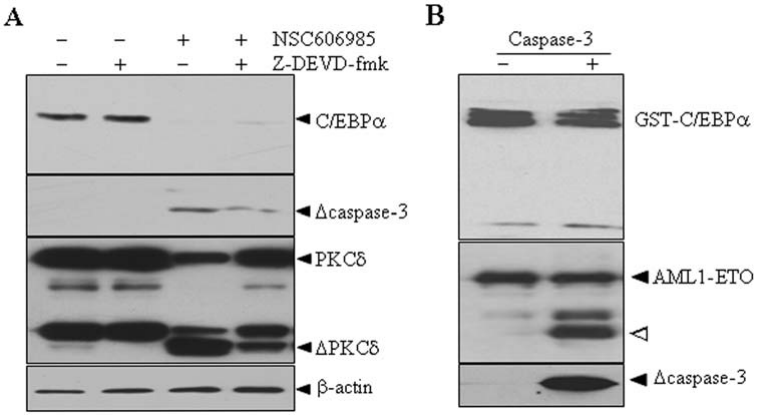
Caspase-3 dose not degrade C/EBPα protein. (A) After pre-incubation with 40 µM Z-DEVD-fmk for 1 hour, NB4 cells were treated with or without 25 nM NSC606985 for an additional 18 hours. Then, the indicated proteins were detected by Western blots. Δcaspase-3 and ΔPKCδ indicate activated fragments of caspase-3 and cleaved catalytic fragment of PKCδ, respectively. (B) Bacteria expressing GST-C/EBPα or GST-AML1-ETO were incubated with or without recombinant his-tagged active fragment of caspase-3 at 25°C for 3 hours. The mixtures were blotted with anti-C/EBPα, ETO and/or activated capase-3 antibodies. “Δ” points to a cleaved fragment of AML1-ETO protein.

Considering that all five apoptosis insults used here (Figure 1) and the inducible expression of PKCδ-CF (Figure 3E) could activate caspase-3 and that inhibition of PKCδ activation by rottlerin and DN-PKCδ expression abrogated caspase-3 activation (Figure 3A/C), we asked whether PKCδ mediates degradation of C/EBPα protein via activated caspase-3, which is an important member of a unique caspase family of conserved cysteine proteases that cleaves an impressive array of substrates after an aspartate residue [28]. As reported before [14], the pretreatment of cell-permeable caspase-3 inhibitor Z-DEVD-fmk almost completely blocked caspase-3 activation, which in turn partially inhibited proteolytic cleavage of PKCδ (Figure 4A) and it also partially blocked apoptosis induced by NSC606985 (data not shown). However, this inhibitor failed to rescue NSC606985-reduced C/EBPα protein (Figure 4A). By the way, the purified recombinant his-tagged active fragment of caspase-3 could cleave AML1-ETO protein as described [29], [30], but it did not destroy the GST-C/EBPα protein (Figure 4B). All these experiments indicated that activated caspase-3 does not contribute to the degradation of C/EBPα protein during apoptosis.

### Proteasome inhibitors antagonize increased degradation of C/EBPα protein during apoptosis

**Figure 5:**
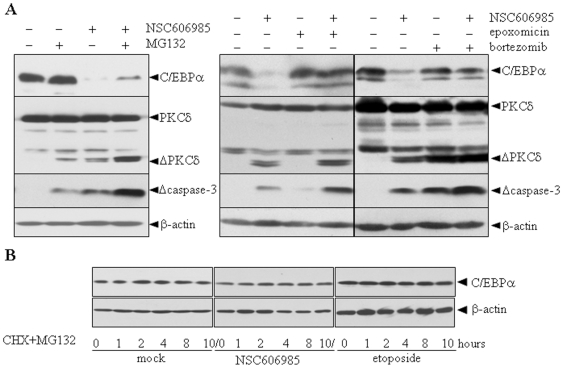
Proteasomal pathway involves increased C/EBPα protein degradation induced by proteolytically activated PKCδ. (A) U937 cells were treated with or without 50 nM NSC606985 for 36 hours in the presence and absence of 10 µM of MG132 for 5 hours, 1 µM of epoxomicin for 5 hours or 1 µM bortezomib for 12 hours respectively before harvest. C/EBPα, Δcaspase-3 and PKCδ proteins were detected by Western blots with β-actin as an internal control. (B) After pre-treatment with or without 25 nM of NSC606985 or 0.5 µM of etoposide for 12 hours, NB4 cells were incubated with 10 µM MG132 for 30 min prior to the addition of 10 µg/ml CHX for the indicated hours. C/EBPα protein was detected with β-actin as a loading control.

Since C/EBPα protein can be degraded via the ubiquitin-proteasomal system [31], we continued to explore the possible contribution of the system in PKCδ-induced C/EBPα degradation. For this purpose, three specific proteasome inhibitors including epoxomicin [32], bortezomib [33] and MG-132 [34] were each used to treat U937 cells together with 50 nM of NSC606985. The results showed that all three inhibitors, especially epoxomicin and bortezomib, significantly antagonized NSC606985-induced decrease of C/EBPα protein, although MG132 and bortezomib at concentration used also induced and/or enhanced NSC606985-induced PKCδ and caspase-3 activation (Figure 5A) and cell death (Figure S5) due to their potential cell toxicities widely documented [35]. Furthermore, MG132 also inhibited degradation of C/EBPα protein in the presence of CHX in NSC606985/etopsoside-treated or untreated NB4 cells (Figure 5B). These results suggested that the proteasomal mechanism contributes to the enhanced degradation of C/EBPα protein upon apoptosis.

### The activated PKCδ possibly mediates the enhanced ubiquitination of C/EBPα protein during apoptosis induction

**Figure 6:**
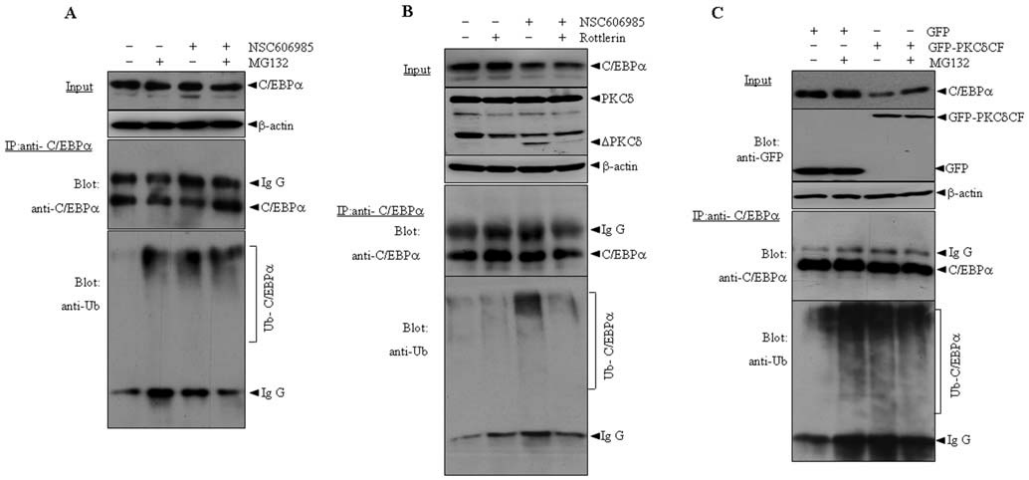
Ectopic expression of active form of PKCδ facilitates ubiquitination of C/EBPα protein. (A) NB4 cells were treated with or without 25 nM of NSC606985 for 12 hours and/or 10 µM MG132 for 5 hours before harvest. (B) After pretreatment for 2 hours in the presence or absence of 1 µM of rottlerin, NB4 cells were treated with or without 25 nM of NSC606985 for additional 12 hours. (C) HEK293T cells were transfected with C/EBPα, ubiquitin and other plasmids as indicated for 24 hours. Then, cells were treated with or without 20 µM MG132 for 5 hours before harvest. Cell lysates were co-immunoprecipitated with anti-C/EBPα antibody, and precipitates or total lysate (input) were detected by western blots for GFP, PKCδ, C/EBPα and ubiquitin.

Next we tested whether NSC606985 induces the ubiquitination of C/EBPα protein. To do this, NB4 cells were treated with or without 25 nM of NSC606985 and/or proteosome inhibitor MG132 for 12 hours, at which point PKCδ was activated without decrease of C/EBPα protein (Figure 1A/6A), and C/EBPα protein was immunoprecipitated followed by western blot with antibody against ubiquitin. As shown in Figure 6A, MG132 treatment effectively increased the ubiquitinated C/EBPα protein which could not be detected in untreated cells, indicating the specificity and effectiveness of the assay system. NSC606985 treatment also significantly increased the ubiquitinated C/EBPα protein regardless of the presence of MG132. More intriguingly, when the activation of PKCδ was blocked by rottlerin, as shown in Figure 6B, the enhanced ubiquitination of C/EBPα protein also disappeared during NSC606985 treatment. Furthermore, GFP-tagged PKCδ-CF or its empty vector pEGFP-N1 was transfected into HEK293T cells together with C/EBPα and His6-tagged ubiquitin constructs, followed by immunoprecipitation of C/EBPα protein and blots for the ubiquitin and C/EBPα proteins. The results demonstrated that only co-transfection of C/EBPα and His6-ubiquitin induced a lower degree of ubiquitination of C/EBPα protein (lane 1, Figure 6C), which was significantly enhanced by MG132 treatment (lane 2, Figure 6C). Similar to that seen in NSC606985-treated NB4 cells (Figure 6A/B), addition of GFP-PKCδ-CF also increased the ubiquitinated C/EBPα protein in the presence of co-transfection of C/EBPα and ubiquitin (lane 3, Figure 6C). It should be pointed out that, although the transfected C/EBPα protein could also be destructed by the ectopically expressed PKCδ-CF and rescued by MG132 (top panel, Figure 6C), co-administration of MG132 did not significantly increase the ubiquitinated C/EBPα protein induced by co-transfection of PKCδ-CF and ubiquitin (lane 3, Figure 6C) or NSC606985 treatment (Figure 6A). All these results suggested that the activated PKCδ mediated the enhanced ubiquitination of C/EBPα protein under NSC606985 treatment.

### Silencing of C/EBPα expression by siRNAs enhances while inducible expression of C/EBPα inhibits NSC606985/etoposide-induced apoptosis in leukemic cells

**Figure 7:**
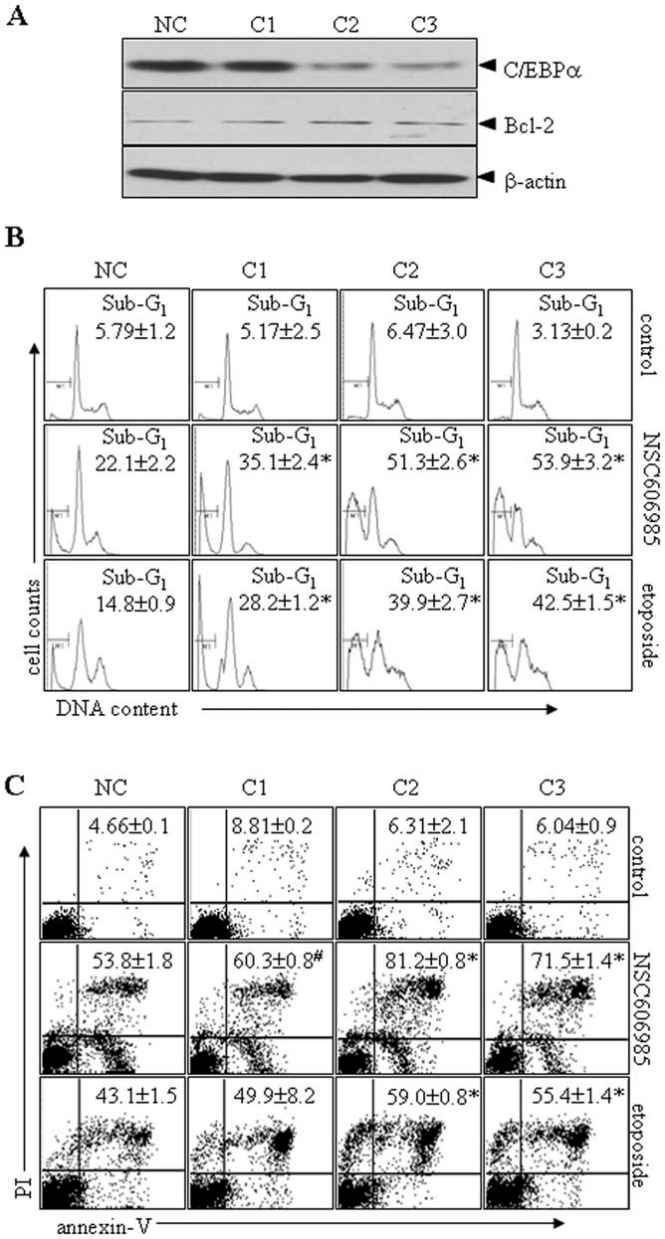
Silencing of C/EBPα expression by siRNAs enhances NSC606985/etoposide-induced apoptosis in leukemic cells. (A) U937 cells were stably transfected with siRNA C1–3 against C/EBPα or negative control vector (NC), and C/EBPα and Bcl-2 proteins were blotted with β-actin as a loading control. (B) U937 cells with stable transfections of C1-C3 or NC were treated with 2 µM etoposide or 200 nM NSC606985 for 24 hours, and apoptotic sub-G_1_ cells% were determined on flow cytometry. (C) U937 cells with stable transfections of C1-C3 or NC were treated with 2 µM etoposide or 200 nM NSC606985 for 36 hours, and annexin-V^+^ cells% were determined on flow cytometry. The values represent mean±S.D. of triplicates in an independent experiment, which was repeated more than three times with the same results. The symbols * and # represent P<0.01 and <0.05 compared with NC cells with the corresponding treatment.

**Figure 8:**
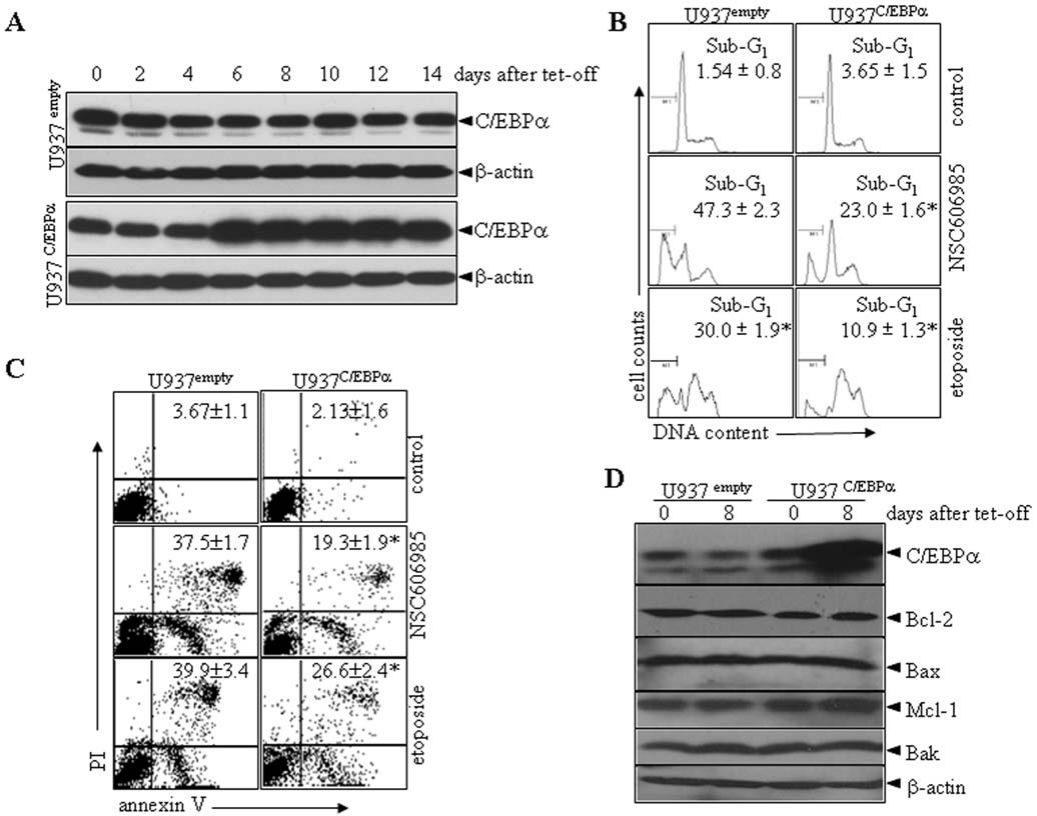
Inducible C/EBPα expression inhibits NSC606985/etoposide-induced apoptosis in leukemic cells. (A) U937^empty^ and U937^C/EBPα^ cells were incubated for days as indicated after removal of tetracycline (Tet), and C/EBPα protein was detected with β-actin as a loading control. (B-C) After pre-incubation in the absence of tetracycline for 8 days, U937^empty^ and U937^C/EBPα^ cells were treated with 200 nM NSC606985 or 2 µM etoposide for 24 hours, and the percentages of apoptotic sub-G_1_ cells (B) and annexin-V^+^ cells (C) were determined on flow cytometry. The values represent mean±S.D. of triplicates in an independent experiment, which was repeated more than three times with the same results. The symbol * represents P<0.01 compared with U937^empty^ cells with the corresponding treatment. (D) U937^empty^ and U937^C/EBPα^ cells were incubated for days as indicated after removal of tetracycline and the indicated proteins were detected with β-actin as a loading control.

Finally, three pairs of siRNAs (C1–3) against C/EBPα were designed and transfected into U937 cells. With selection by G418, C2 and C3 siRNAs but not C1 siRNA significantly inhibited C/EBPα protein expression, compared with negative control (Figure 7A). The suppression of C/EBPα expression by C2 and C3 siRNAs statistically significantly enhanced NSC606985/etoposide-induced activation of caspase-3 (Figure S6) and apoptosis, the latter being determined by the percentages of sub-G_1_ cells (Figure 7B), annexin-V^+^ cells (Figure 7C) as well as cell morphology (Figure S7). On the other hand, an inducible C/EBPα-expressing cell line (U937^C/EBPα^) was generated using myeloid leukemic U937T cells as described above. U937^empty^ cell line with transfection of empty vector was used as a control. As can be visualized in Figure 8A, C/EBPα protein was significantly induced at 6 days after tetracycline withdrawal in U937^C/EBPα^ cells. Hence, we treated U937^empty^ and U937^C/EBPα^ cells with NSC606985 or etoposide when tetracycline was removed for 8 days. The results showed that the inducible expression of C/EBPα partially inhibited NSC606985/etoposide-induced caspase-3 action (Figure S8) and apoptosis to a statistic degree (Figure 8B/C and Figure S8). These results supported the anti-apoptotic role of C/EBPα. Additionally, conditional expression of C/EBPα protein failed to alter expressions of apoptosis-related Bcl-2, Bak, Bax and Mcl-1 genes in U937^C/EBPα^ cells (Figure 8D), and the suppression of C/EBPα expression by siRNAs also failed to alter Bcl-2 protein level (Figure 7A).

## Discussion

Previously, a variety of DNA-damaging agents such as UV, etoposide and bleomycin were shown to induce C/EBPα expression in keratinocytes, but not in HepG_2_, NRK or NIH 3T3 cells, indicating that the induction of C/EBPα by DNA damage is dependent upon cell context [36]. Although we also found that both NSC606985 and etoposide appeared to induce a rapid but transitory elevation of CEBPA expression in U937 but not NB4 cells, all apoptosis-inducing agents tested here induced a profound down-regulation of C/EBPα protein in leukemic cell lines NB4 and U937 as well as in primary leukemic cells when apoptosis occurred. Further investigation showed that NSC606985 and etoposide significantly shortened the half-life of C/EBPα protein and reduced expression of CEBPA mRNA and CEBPA promoter-driven luciferase reporter assay, suggesting that inhibition of transcription and induction of protein degradation of C/EBPα contributed to down-regulation of C/EBPα protein during apoptosis induction.

Inhibition of caspase-3 by Z-DEVD-fmk failed to rescue NSC606985-reduced C/EBPα protein, and the purified recombinant active fragment of caspase-3 did not cleave C/EBPα protein *in vitro*, indicating that activated caspase-3 does not contribute to PKCδ-induced instability of C/EBPα protein during apoptosis. It is believed that PKCδ is a substrate for the apoptosis-critical executioner caspase-3, which cleaves PKCδ to a 41 kDa catalytically active fragment [19], [37]. Vice versa, the ability of PKCδ activation to enhance DNA-damaging agents such as cisplatin-induced apoptosis was correlated with their ability to enhance caspase-3 activation, and rottlerin inhibits cisplatin or NSC606985-induced activation of caspase-3 [14], [38], [39], proposing that PKCδ also acts upstream of caspase-3 to regulate its activation. Therefore, there is a feedback loop between PKCδ and caspase-3 to promote apoptosis [18], [40], [41]. Of note, it remains not to be completely clear how DNA damage activates PKCδ. Although it was proposed that PKCδ may regulate its own cleavage in response to apoptotic stimuli [38], recently we used quantitative proteomic analysis to identify the differentially expressed proteins before PKCδ activation induced by NSC606985 in U937 cells. As a result, a total of 33 proteins were found to be dysregulated in this situation and we proposed that the downregulation of N-myc downstream regulated gene 1 (NDRG1) is involved in proteolytic activation of PKCδ during apoptosis induction [39].

Next, we investigated whether the down-regulation of C/EBPα protein is mediated by proteolytically activated PKCδ. Our results showed that inhibition of action of PKCδ by rottlerin and stable transfection of DN-PKCδ partially rescured C/EBPα protein but not its mRNA level upon NSC606985 treatment, suggesting that the activated PKCδ protein contributes to the increased degradation of C/EBPα protein but not to the reduced transcription of CEBPA by DNA-damaging agents. This notion is also supported by the fact that transfected expression of the PKCδ-CF reduces C/EBPα protein but not its mRNA.

In accordance with previous reports [14], [42], [43], overexpression of the PKCδ-CF is sufficient to induce apoptosis, and proteolytic activation of PKCδ exerts a critical role in NSC606985-induced apoptosis at least in AML cells. However, mechanisms of PKCδ-mediated apoptosis are controversial. PKCδ is a mobile enzyme, and its intracellular distribution is altered upon activation. This is one way to reach the appropriate targets in order to elicit its biological responses. One of the most important questions is how and where PKCδ transduces its apoptotic signals. It appears that PKCδ can act at several locations for apoptosis induction, including the plasma membrane, mitochondria and nucleus. Previously, we showed that NSC606985-induced PKCδ cleavage simultaneously occurs in the cytoplasm and nuclei of both NB4 and U937 cells [14]. In spite of the subcellular localization, it remains to be illustrated what are the downstream targets of PKCδ for apoptosis induction. In response to DNA damage, as reported [44], catalytic fragment of PKCδ can phosphorylate and inactivate DNA-dependent protein kinase, resulting in further cell death. PKCδ can also phosphorylate p73β, a member of the p53 family [45]. Lamin B and phospholipid scramblase 3 were also proposed to be substrates of PKCδ and could contribute to apoptosis [46], [47]. Thus, PKCδ can contribute to apoptosis by phosphorylating different targets that are localized in the nucleus, plasma membrane or mitochondria. However, our immunoprecipitation experiment did not support the C/EBPα-PKCδ interaction and the activated PKCδ did not appear to directly induce phosphorylation of C/EBPα protein (data not shown). On the other hand, the NSC606985-induced decrease of C/EBPα protein was significantly antagonized by specific proteasome inhibitors MG-132, epoxomicin and bortezomib. Of great importance, NSC606985 and ectopic expression of PKCδ-CF significantly increased ubiquitination of endogenously and ectopically expressed C/EBPα protein, while rottlerin significantly inhibited the enhanced ubiquitination of endogenously expressed C/EBPα protein during NSC606985 treatment. Although the effect of the forced expression of DN-PKCδ on ubiquitination of C/EBPα protein remains to be determined, all these results suggested that activated PKCδ mediates proteasome-dependent degradation of C/EBPα protein, which is involved the enhanced degradation of C/EBPα protein upon apoptosis induction. In fact, a recent report showed that the von Hippel-Lindau tumor-suppressor protein, a ubiquitin-ligase (E3) directing proteasome-dependent degradation of targeted proteins, interacts directly with the catalytic domain of PKCδ in cells [48], and PKCδ does mediate proteasome-dependent degradation of certain proteins such as mitogen-activated protein kinase phosphatase-1 and p21^cip1^ [49], [50]. By the way, we also found that activated PKCδ could alter the subcellular localization of C/EBPα protein (data not shown), which might be related to the degradation of C/EBPα protein.

It remains to be investigated how CEBPA mRNA was reduced during apoptosis induction. The actinomycin-inhibiting test showed that NSC606985 or etoposide treatment failed to alter the stability of CEBPA mRNA (data not shown), suggesting that the transcription of CEBPA may be regulated, which could be supported by the CEBPA promoter driven luciferase reporter assay. As reported [51]–[53], the consensus recognition sequences of some apoptosis-related transcription factors such as sp1, myc/max and AP2α are present in the promoter region of CEBPA gene. Therefore, the down-regulation of CEBPA during apoptosis induction may also be associated with these factors. Additionally, the auto-activation function of C/EBPα protein has been reported [54]. Thus, we cannot exclude the possibility that the decrease of CEBPA mRNA is due to the reduced C/EBPα protein although the decrease of C/EBPα protein but not its mRNA was partially rescued by rottlerin and the expression of PKCδ-CF also reduced C/EBPα protein but not its mRNA.

The inhibition of caspase-3 by its specific inhibitor did not completely inhibit PKCδ activation-related apoptosis, suggesting that caspase-3-independent mechanism(s) also contributes to the event [14], [18], [42], [55]. The fact that C/EBPα protein was one of the down-stream effectors of the activated PKCδ during apoptosis induction pushed us to explore its possible role in apoptotic event. Here we showed that inhibition of C/EBPα expression enhanced, while its over-expression inhibited, DNA-damaging agents-induced apoptosis, which could be shown by annexin-V^+^ cells%, especially by sub-G_1_ cells% and apoptosis-related morphologic analysis. These results demonstrated that the modulation of C/EBPα expression contributes, at least partially, to the activation of PKCδ-mediated apoptosis. It was proposed that C/EBPα induces the expression of the anti-apoptotic Bcl-2 gene [11]. However, this notion could not be confirmed in this work. Similarly, conditional expression of C/EBPα protein did not alter other apoptosis-related genes, including Bak, Bax and Mcl-1. Therefore, molecular mechanisms of anti-apoptotic effect of C/EBPα remain to be explored.

Taken together, our results demonstrated that C/EBPα expression is down-regulated in apoptotic leukemic cells partially through induction of protein degradation, the latter involving proteolytically activated PKCδ-enhanced ubiquitination of C/EBPα protein. Furthermore, C/EBPα exerts a role in chemotherapeutic drugs-induced apoptosis. These results would shed novel sights for understanding mechanisms of PKCδ-related apoptosis and functions of C/EBPα between differentiation and apoptosis.

## Materials and Methods

### Cell treatment and apoptosis assay

Leukemic cell lines, including NB4 [56] and U937, were grown in RPMI-1640 medium (Sigma-Aldrich, St Louis, MO) supplemented with 10% heat-inactivated fetal bovine serum (FBS, Gibco BRL, Gaithersburg, ML) in 5% CO_2_/95% air humidified atmosphere at 37°C. U937T cells, which were kindly provided by Dr. Tenen D.G. at the Harvard Medical School (Boston, MA) [27], are U937 cells stably transfected with a pUHD-tTA under the control of a tetracycline-inducible promoter. U937T cells were cultured in RPMI-1640 medium supplemented with 1 µg/ml tetracycline (Sigma-Aldrich) and 0.5 µg/ml puromycin (Sigma-Aldrich) with 10% FBS. For apoptosis induction, about 2×10^5^ cells/ml were initially seeded and incubated with indicated concentrations of NSC606985 [kindly provided by National Cancer Institute Anticancer Drug Screen standard agent database and dissolved in double distilled water (ddH_2_O) as a 1 mM stock solution], etoposide [BIOMOL, Plymouth, PA, dissolved in dimethyl sulfoxide (DMSO) as 20 mM stock solution], doxorubicin (BIOMOL, dissolved in ddH_2_O as 20 mM stock solution) or As_2_O_3_ (Sigma-Aldrich), in the presence or absence of rottlerin (BIOMOL) prepared in ethanol as 1 mM stock solution or Z-DEVD-fluoromethyl ketone (Z-DEVD-fmk, BD Biosciences, San Diego, CA) dissolved in DMSO. MG132 and epoxomicin (BIOMOL) were dissolved in DMSO or bortezomib (Millennium Predictive Medicine Inc., Cambridge, MA) in phosphate-buffered saline (PBS) and stored at −20°C until usage. For induction of apoptosis by UV irradiation, cells were exposed to a germicidal lamp providing predominantly 254-nm UV-C light (Philips TUV G30T8 30 W bulb). To assess apoptosis, cell morphology, cell viability, annexin V with and without PI staining and sub-G_1_ cells as previously described [14] as well as activity of caspase-3 were measured. The activity of caspase-3 was measured by caspase-3 assay kit colorimetric (sigma-aldrich) according to the manufacturer's instruction.

### Primary cells from patients and ethics statement

Mononuclear cells in BM, which were obtained from one case of M3-subtyped and one case of M5-subtyped AML patients according to French-American-British classification, were aspirated by Ficoll-Paque liquid and suspended in RPMI-1640 medium with 10% FBS. This study was conducted according to the principles expressed in the Declaration of Helsinki. The study was approved by the Ethic Committee of Shanghai Jiao Tong University School of Medicine. All patients provided written consent for the collection of samples and subsequent analysis.

### Plasmids

Full-length C/EBPα cDNA and PKCδ-CF (the catalytic fragment of PKCδ) were amplified by PCR from pCMV-SPORT-C/EBPα plasmid (kindly provided by Dr Gombart AF in Cedars-Sinai Medical Center, Los Angeles, CA) and pEGFP-PKCδ-CF (kindly provided by Dr Reyland ME in School of Dentistry, University of Colorado Health Sciences Center, Denver, CO), respectively, and were subcloned into pTRE2hyg expression vector (BD clontech, Palo Alto, CA) to form the pTRE2hyg-C/EBPα or pTRE2hyg-PKCδ-CF plasmid. Human C/EBPα or human AML1-ETO cDNA amplified from U937-A/E 9/14/18 cells by RT-PCR [57] was also subcloned into pGEX-4T3 vector (Amersham Biosciences, Buckinghamshire, England) to construct GST-tagged C/EBPα or AML1-ETO-expressing vector. To obtain His-tagged active caspase-3, human caspase-3 cDNA lacking N-terminal 28 amino acid residues was amplified from U937 cells and subcloned into pQE30 (Qiagen, Valencia, CA) vector following the protocol of Lee KK et al [58]. Plasmid construct PMT107 (vector for His6-Ubiquitin expression) was a kind gift from Dr. Ying Jin in institute of Health Science, SIBS (Shanghai, CHINA) [59]. DN-PKCδ was amplified by PCR from pCDNA3-PKC-DNδ plasmid [a gift from Dr.Jae-Won Soh (Inha University, Incheon, Korea) [60] and was subcloned into a puromycin murine stem cell provirus (pMscv-puro) expression vector (Clontech) to form the pMscv-puro-Flag-DN-PKCδ plasmid. The sequences of all cDNA inserts of plasmids were confirmed by sequencing.

### Luciferase assay

pRL-SV40 vector and CEBPA promotor-driven luciferase plasmid, which was a pGL3-basic-luciferase reporter vector (Promega, Madison, WI) cloned with a 3.1 kb DNA fragment consisting the 5′ flanking region (−1 to −3000) and the first 103 bp of the first exon of the human CEBPA gene (GenBank NM_004364), were transferred into NB4 cells by electroporation using Gene-Pulser II (Bio-Rad, Hercules, CA) at 150 V and 960 µF or U937 cells by using Lipofectamine 2000 (Invitrogen, Carlsbad, CA) following the manufacturer's protocol. Twenty-four hours after transfection, NB4 and U937 cells were treated with or without NSC606985 or etoposide for hours as indicated. The cell luciferase activity was measured by the Dual-Luciferase Assay system (Promega) according to the manufacturer's instructions. The measured luciferase activity was normalized for pRL-SV40 Renilla luciferase activity for each sample, and luciferase activity was expressed as folds over the corresponding untreated cells respectively.

### Establishment of stable transformants

To generate U937^C/EBPα^ or U937^PKCδ-CF^ stable transformants, 1×10^7^ U937T cells were washed in RPMI 1640 medium and resuspended in 0.2 ml of Isceve's Modified Dulbecco's medium without FBS. Twenty micrograms of pTRE2hyg C/EBPα or pTRE2hyg PKCδ-CF plasmid in 20 µl ddH_2_O was transferred to electroporation cuvette with a 0.4 cm gap (Bio-Rad, Hercules, CA). Electroporation was performed using a Gene-Pulser II (Bio-Rad) at 170 V and 960 µF. The samples were then transferred to complete RPMI-1640 medium. Twenty-four hours later, 1 µg/ml tetracycline, 0.5 µg/ml puromycin and 500 µg/ml hygromycin B (Clontech) were added. Cells were then incubated at 37°C in 5% CO_2_. Positive polyclonal populations were identified based on Western blot after tetracycline removal, and were maintained in RPMI-1640 medium supplemented with 10% FBS, and 1 µg tetracycline, 0.5 µg/ml puromycin and 500 µg/ml hygromycin B. For retroviruses transfection, viral supernatants were produced in HEK293T cells cotransfected with the pMSCV-puro or pMscv-puro-Flag-PKC-DNδ constructs and packaging vectors pEQPAM (containing gag-pol, provided by Dr. Lishan Su in UNC Chapel Hill, USA) and VSV-G (Clontech). Viral supernatants were collected 48 hours after transfection, filter-sterilized, and stored at −80°C. Before using 6-well plate was coated with 10 µg/ml Fibronectin for more than 4 hours at 37°C, and the viral supernatants with final concentration of 4 µg/ml Polybrene were added to U937 cells 2×10^5^ cells/well in 6-well plate. Forty eight hours later, selection with puromycin (0.7 µg/ml) was started. Positive polyclonal populations were identified based on Western blot for Flag-DN-PKCδ.

### siRNA design and transfection

Three pairs of complementary siRNA oligonucleotides against C/EBPα (C1–3) were synthesized by Invitrogen (Shanghai, China), annealed and ligated into pSilencer 3.1-H1-neo vector (Ambion, Austin, TX). Their target sequences for C/EBPα were 5′-GAACAGCAACGA GTACCGG-3′ for C1, 5′-CCTTGTGCCTTGGAAATGC-3′ for C2 and 5′-CACTTGTATCTGGCC TCTG-3′ for C3. These siRNA vectors and the negative control pSilencer neo vector (Ambion) were respectively transfected into U937 cells using the Gene-Pulser II (Bio-Rad) with square-wave electroporation of 2 pulses, 0.18 kV, 25 ms, 1 Hz. Forty-eight hours later, 800 µg/ml G418 (Calbiochem) was added to the medium and the stable transformants were selected by testing for C/EBPα protein.

### Purification of recombinant caspase-3 and *in vitro* proteolysis of C/EBPα protein

His-tagged caspase-3 protein was expressed in bacteria BL21 (DE3) by induction with 1 mM isopropylthiogalactopyranoside (IPTG) at 30°C, and was purified by affinity chromatography on Ni-NTA-agarose (Qiagen). The GST-C/EBPα or GST-AML1-ETO was expressed in BL21 by induction with IPTG at 28°C and purified using Bulk and RediPack GST Purification Modules (Amersham Biosciences). Purified GST-C/EBPα or GST-AML1-ETO protein was incubated with purified recombinant his-tagged active fragment of caspase-3 in 100 mM HEPES (pH 7.2) containing 10 mM dithiothreitol and 10% (v/v) glycerol at 25°C for 180 min. The reaction was stopped by the addition of an equal volume of SDS-PAGE (sodium dodecyl sulfate polyacrylamide gel electrophoresis) sample buffer and was then subjected to Western blot.

### RT-PCR

Total cellular RNA was extracted by TRIzol reagent (Invitrogen), followed by treatment with RNase-free DNase (Promega). RT was performed with a cDNA synthesis kit according to the manufacturer's instructions (Applied Biosystem, Forster City, CA). For real-time quantitative RT-PCR, the following specific oligonucleotide primers were used for C/EBPα (sense, 5′-GAATCTCCTAGTCCTGGCTC-3′, antisense, 5′-GATGAGAACAGCAACGAGTAC-3′ ) with 18S as an internal control (sense, 5′-AGGCCCTGTAATTGGAATGAGTC-3′, antisense, 5′- GCTCCCAAGATCCAACTACGAG-3′). Real-time RT-PCR was performed and data were analyzed as reported previously [61].

### Cycloheximide inhibition test

NB4 cells were pretreated with 25 nM NSC606985 or 0.5 µM etoposide for 12 hours. Thereafter, cells were further incubated with 10 µg/ml cycloheximide (CHX, Sigma-Aldrich) as indicated in the figure legend. C/EBPα protein was tested by Western blots using β-actin as loading controls.

### Ubiquitination detection and western blot

To detect the ubiquitination of C/EBPα protein, cell lysates were immediately boiled in 1% SDS for 15 min followed by dilution with the lysis buffer (50 mM HEPES, 50 mM NaCl, 0.1% Tween-20, 10% glycerol, 20 mM sodium pyrophosphate, 1 mM dithiothreitol, plus protease inhibitors) to 0.1% SDS. Then, the lysates were incubated with anti-C/EBPα antibody (sc-61, Santa Cruz Biotech, Santa Cruz, CA) and protein A-agarose (Santa Cruz) and rocked overnight at 4°C. The complexes were centrifuged at 1,200 g and washed three times with ice-cold lysis buffer. Immunoprecipitated proteins and protein extracts were equally loaded on 10–12% SDS-polyacrylamide gel, and transferred to nitrocellulose membrane (Amersham Bioscience). The blots were stained with 0.2% Ponceau S red to ensure equal protein loading. After blocking with 5% nonfat milk in PBS, the membranes were incubated with antibodies against C/EBPα (sc-61 or c-18), PARP, pro-caspase-3 (E-8), PKCδ, GFP, Bcl-2, p53, Mcl-1, Bax (Santa Cruz), Bak (Sigma-Aldrich), active caspase-3 [cleaved caspase-3 (Asp175)], ubiquitin (Cell Signaling, Beverly, MA) and β-actin (Calbiochem), followed by horseradish perioxidase (HRP)-linked secondary antibodies (Cell Signaling). Detection was performed using a chemiluminescence phototope-HRP kit (Dako, Carpinteria, CA). When necessary, signal intensity of C/EBPα protein was normalized against β-actin as internal control using GS-800 calibrated imaging density meter (Bio-Rad), and folds of changes were expressed compared with untreated cells.

### Statistical analysis

All experiments were repeated at least for three times with the same results. The Student's t-test was used to compare the difference between two different groups. A value of p<0.05 was considered to be statistically significant.

## Supporting Information

Text S1 Supplemental Materials
(0.03 MB DOC)

Figure S1 NSC606985 induces apoptosis of NB4 and U937 cells. NB4 and U937 cells were treated respectively with 25 nM and 50 nM of NSC606985 for hours as indicated, the percentages of annexin-V^+^ cells with and without PI staining (A) and apoptotic sub-G_1_ cells (B, in the gate of line scale) were determined on flow cytometry, and cell morphology was examined after Wright's staining (C). The values represent mean±S.D. of triplicate in an independent experiment, which was repeated more than three times with the same results.
(7.82 MB DOC)

Figure S2 Etoposide induces apoptosis of NB4 and U937 cells. NB4 and U937 cells were treated respectively with 0.5 µM and 1 µM of etoposide for hours as indicated, the percentages of annexin-V^+^ cells with and without PI staining (A) and apoptotic sub-G_1_ cells (B, in the gate of line scale) were determined on flow cytometry, and cell morphology was examined after Wright's staining (C). The values represent mean±S.D. of triplicate in an independent experiment, which was repeated more than three times with the same results.
(8.53 MB TIF)

Figure S3 Various apoptosis-inducing agents induce NB4 cell apoptosis. NB4 cells were treated with 2 µM As_2_O_3_, 0.5 µM doxorubicin or irradiated by 150J UV for hours as indicated, the percentages of annexin-V^+^ cells with and without PI staining (A) and apoptotic sub-G_1_ cells(B, in the gate of line scale) were determined on flow cytometry, and cell morphology was examined after Wright's staining (C). The values represent mean±S.D. of triplicate in an independent experiment, which was repeated more than three times with the same results.
(4.03 MB TIF)

Figure S4 Effects of DN-PKCδ expression on NSC606985 induced CEBPA transcription inhibition. U937^Flag-DN-PKCδ^ and U937^vehicle^ cells were treated with 50 nM NSC606985 for 36 hours. Then, the relative CEBPA mRNA was determined by real-time quantitative RT-PCR. The columns represent means of change folds of CEBPA mRNA against untreated cells, with the bar as S.D. of three independent experiments.
(1.53 MB TIF)

Figure S5 Effects of the proteasome inhibitors on the cell viability in the absence and presence of NSC606985. U937 cells were treated with (black column) or without (white column) 50 nM NSC606985 for 36 hours, and MG132 (10 µM), epoxomicin (1 µM) and bortezomib (1 µM) were added for 5, 5 and 12 hours respectively before harvest with vehicle as control. Cell viability (top panel) was measured by trypan-blue exclusion assay. Annexin-V^+^ cells% (bottom panel) was measured on flow cytometry. All values represent mean±S.D. of triplicates in an independent experiment. All the experiments were repeated more than three times with the same results.
(5.19 MB TIF)

Figure S6 Effects of suppression of C/EBPα expression by siRNAs on NSC606985/etoposide-induced caspase-3 activation. U937 cells with stable transfections of C1-C3 or NC were treated with 2 µM etoposide or 200 nM NSC606985 for hours as indicated, the caspase-3 activation was measured as described in supplemental materials and methods. The values represent mean±S.D. of triplicate in an independent experiment, which was repeated more than three times with the same results. The symbols * represent P<0.01 compared with NC cells with the corresponding treatment.
(2.27 MB TIF)

Figure S7 U937 cells with stable transfections of C1-C3 or NC were treated with 2 µM etoposide or 200 nM NSC606985 for hours as indicated, cell morphological features were examined under microscope after Wright's staining.
(9.37 MB TIF)

Figure S8 After pre-incubation in the absence of tetracycline for 8 days, U937^empty^ and U937^C/EBP^α cells were treated with 200 nM NSC606985 or 2 µM etoposide for hours as indicated, cell morphological features were examined by microscope after Wright's staining of cells (A), and the caspase-3 activation was measured as described in materials and methods (B). The values represent mean±S.D. of triplicate in an independent experiment, which was repeated more than three times with the same results.
(8.09 MB TIF)
