# Supplementary figures and images for: Protein Kinase Cδ Stimulates Proteasome-Dependent Degradation of C/EBPα during Apoptosis Induction of Leukemic Cells
Source: PLoS One. 2009 Aug 7;4(8):e6552. doi: 10.1371/journal.pone.0006552 (PMC2719015; doi:10.1371/journal.pone.0006552)

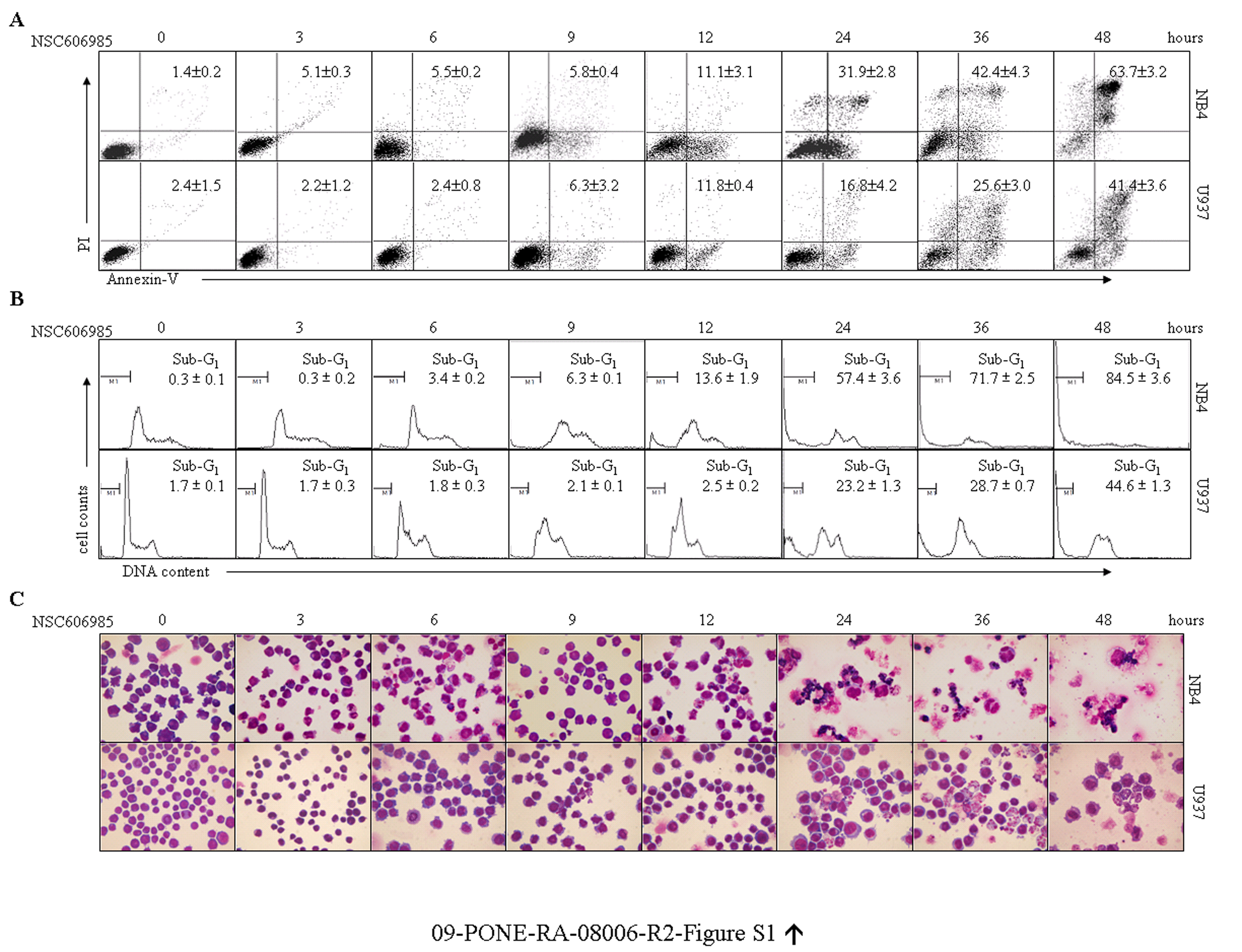

Supplement: Figure S1 — NSC606985 induces apoptosis of NB4 and U937 cells. NB4 and U937 cells were treated respectively with 25 nM and 50 nM of NSC606985 for hours as indicated, the percentages of annexin-V+ cells with and without PI staining (A) and apoptotic sub-G1 cells (B, in the gate of line scale) were determined on flow cytometry, and cell morphology was examined after Wright's staining (C). The values represent mean±S.D. of triplicate in an independent experiment, which was repeated more than three times with the same results. (7.82 MB DOC) [file pone.0006552.s002.tif]

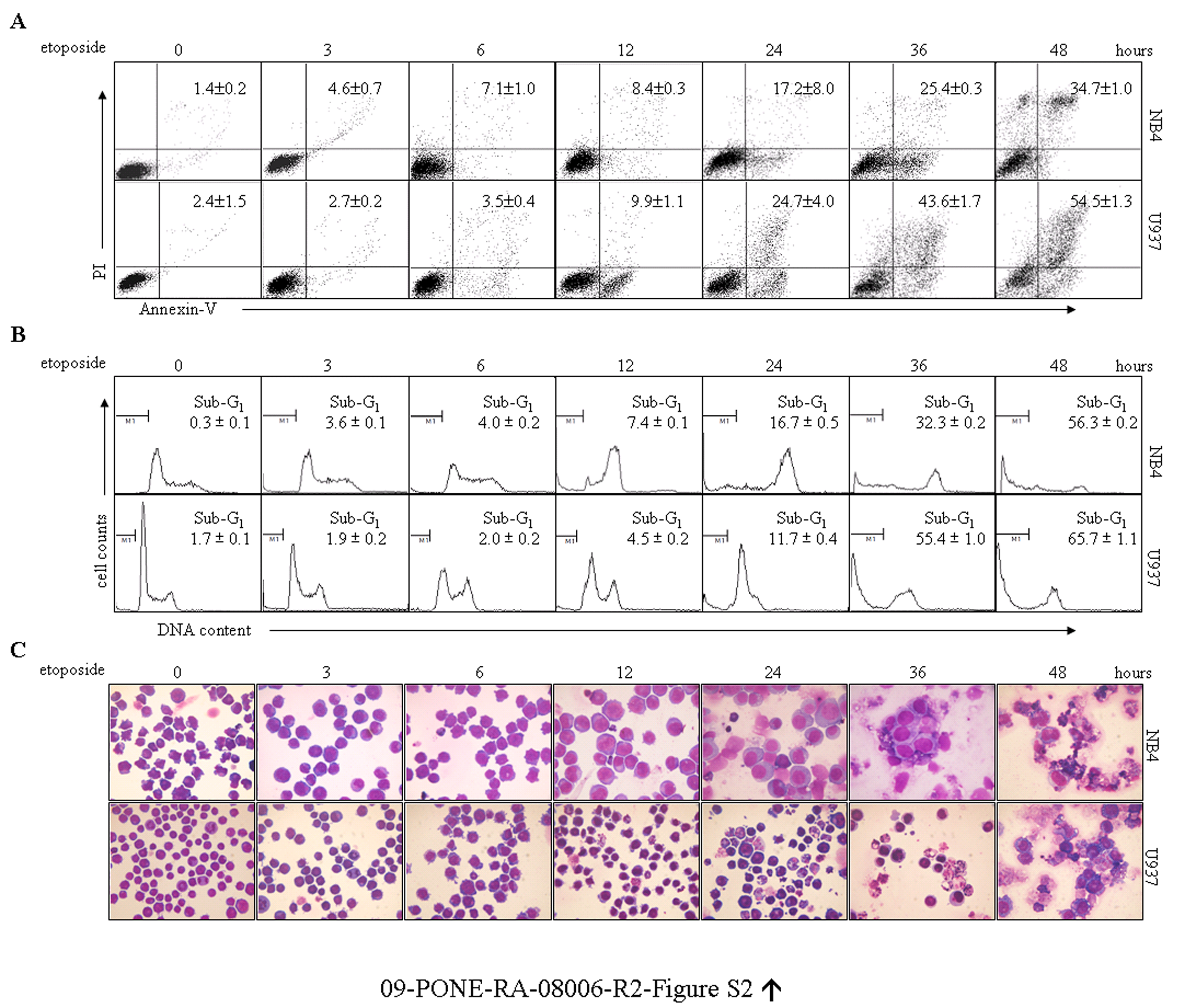

Supplement: Figure S2 — Etoposide induces apoptosis of NB4 and U937 cells. NB4 and U937 cells were treated respectively with 0.5 µM and 1 µM of etoposide for hours as indicated, the percentages of annexin-V+ cells with and without PI staining (A) and apoptotic sub-G1 cells (B, in the gate of line scale) were determined on flow cytometry, and cell morphology was examined after Wright's staining (C). The values represent mean±S.D. of triplicate in an independent experiment, which was repeated more than three times with the same results. (8.53 MB TIF) [file pone.0006552.s003.tif]

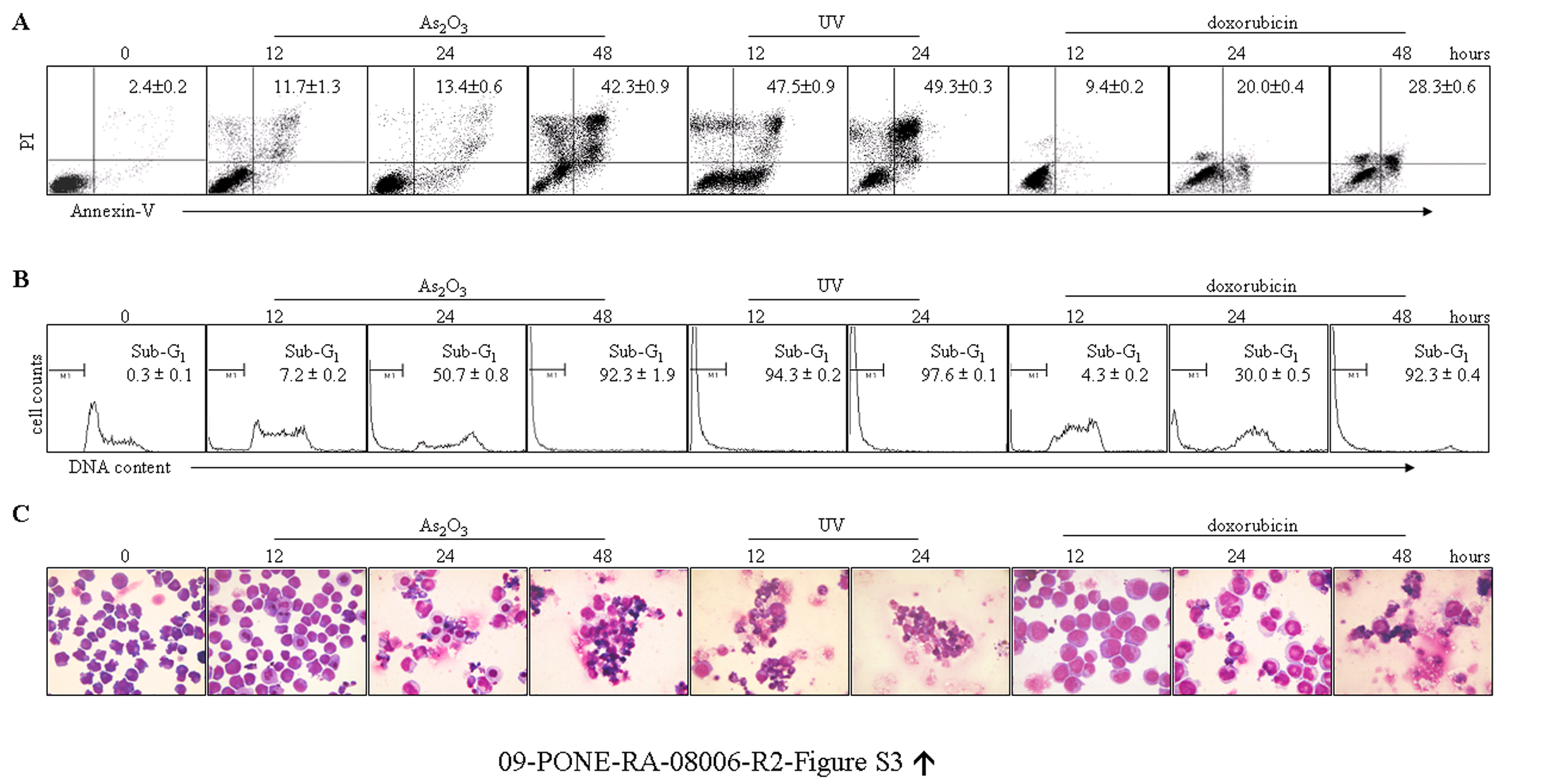

Supplement: Figure S3 — Various apoptosis-inducing agents induce NB4 cell apoptosis. NB4 cells were treated with 2 µM As2O3, 0.5 µM doxorubicin or irradiated by 150J UV for hours as indicated, the percentages of annexin-V+ cells with and without PI staining (A) and apoptotic sub-G1 cells(B, in the gate of line scale) were determined on flow cytometry, and cell morphology was examined after Wright's staining (C). The values represent mean±S.D. of triplicate in an independent experiment, which was repeated more than three times with the same results. (4.03 MB TIF) [file pone.0006552.s004.tif]

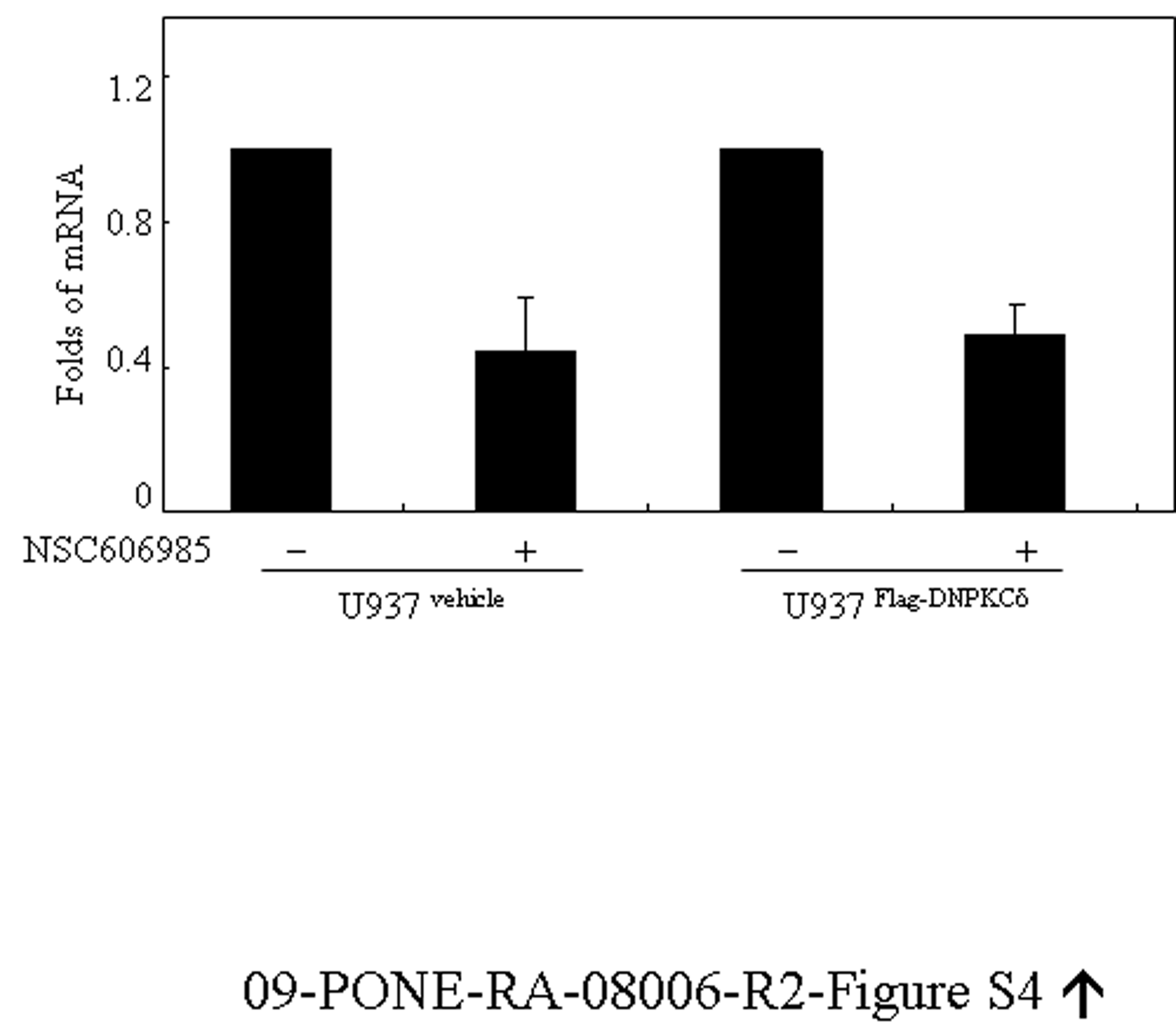

Supplement: Figure S4 — Effects of DN-PKCδ expression on NSC606985 induced CEBPA transcription inhibition. U937Flag-DN-PKCδ and U937vehicle cells were treated with 50 nM NSC606985 for 36 hours. Then, the relative CEBPA mRNA was determined by real-time quantitative RT-PCR. The columns represent means of change folds of CEBPA mRNA against untreated cells, with the bar as S.D. of three independent experiments. (1.53 MB TIF) [file pone.0006552.s005.tif]

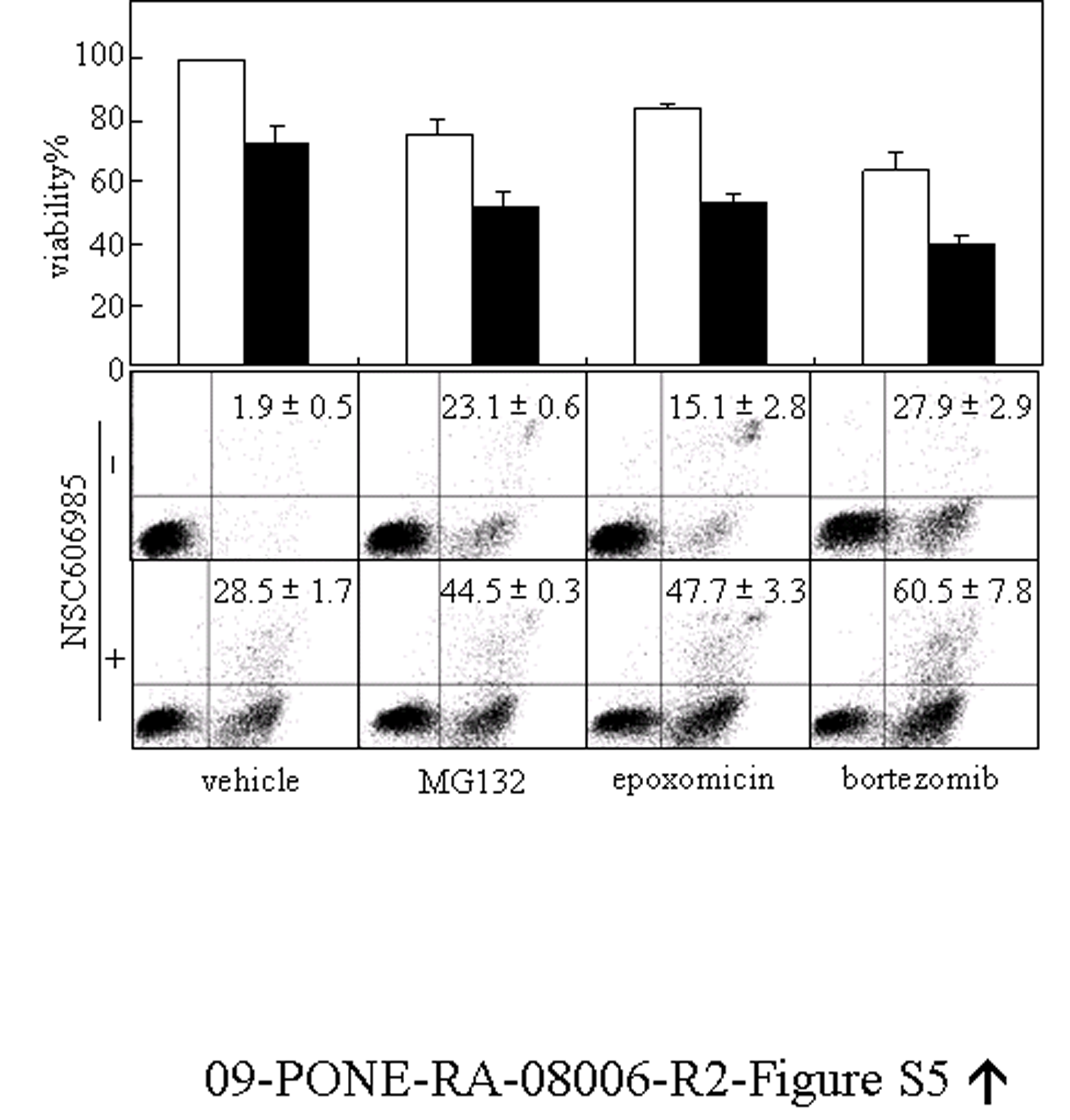

Supplement: Figure S5 — Effects of the proteasome inhibitors on the cell viability in the absence and presence of NSC606985. U937 cells were treated with (black column) or without (white column) 50 nM NSC606985 for 36 hours, and MG132 (10 µM), epoxomicin (1 µM) and bortezomib (1 µM) were added for 5, 5 and 12 hours respectively before harvest with vehicle as control. Cell viability (top panel) was measured by trypan-blue exclusion assay. Annexin-V+ cells% (bottom panel) was measured on flow cytometry. All values represent mean±S.D. of triplicates in an independent experiment. All the experiments were repeated more than three times with the same results. (5.19 MB TIF) [file pone.0006552.s006.tif]

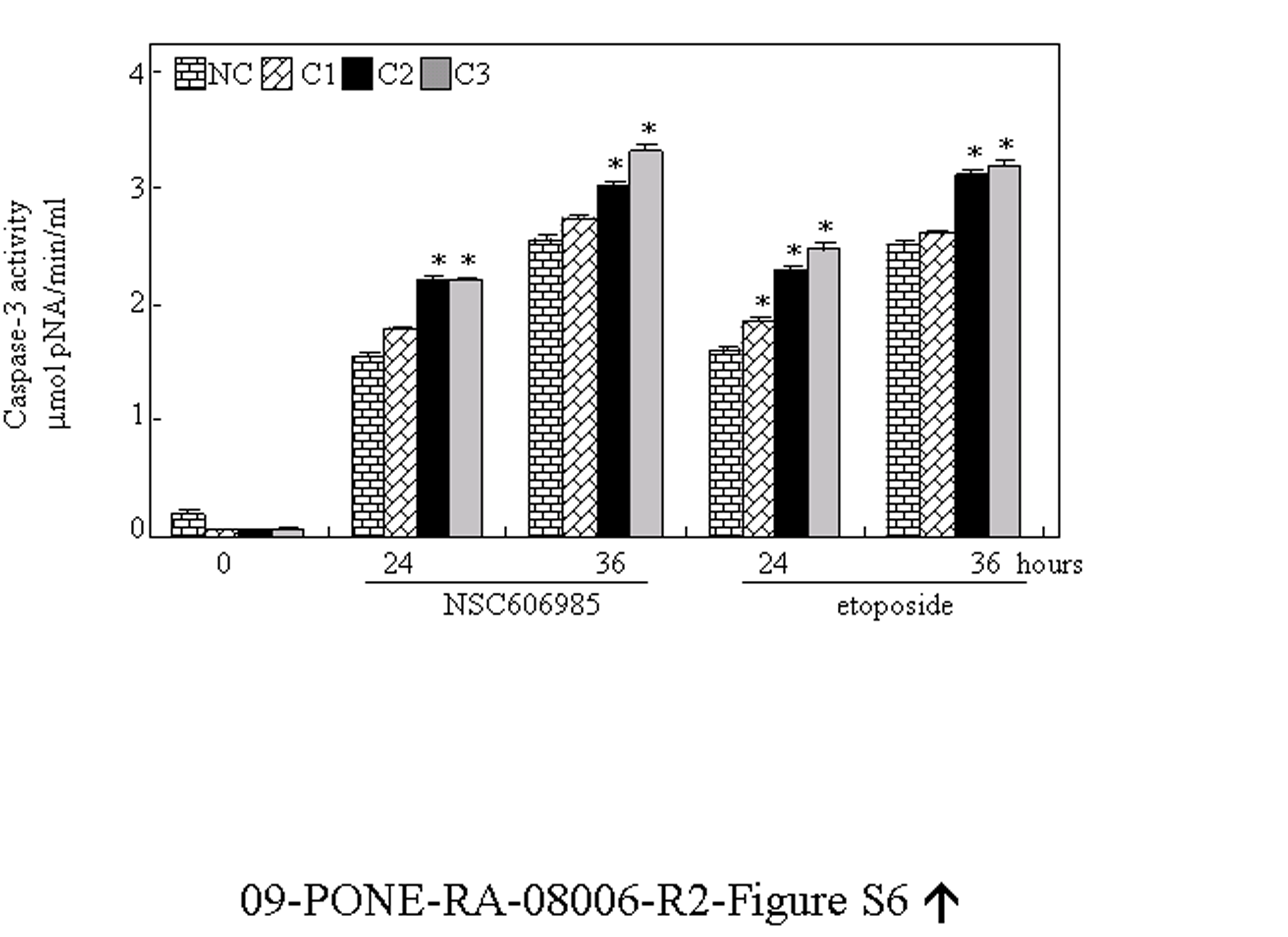

Supplement: Figure S6 — Effects of suppression of C/EBPα expression by siRNAs on NSC606985/etoposide-induced caspase-3 activation. U937 cells with stable transfections of C1-C3 or NC were treated with 2 µM etoposide or 200 nM NSC606985 for hours as indicated, the caspase-3 activation was measured as described in supplemental materials and methods. The values represent mean±S.D. of triplicate in an independent experiment, which was repeated more than three times with the same results. The symbols * represent P<0.01 compared with NC cells with the corresponding treatment. (2.27 MB TIF) [file pone.0006552.s007.tif]

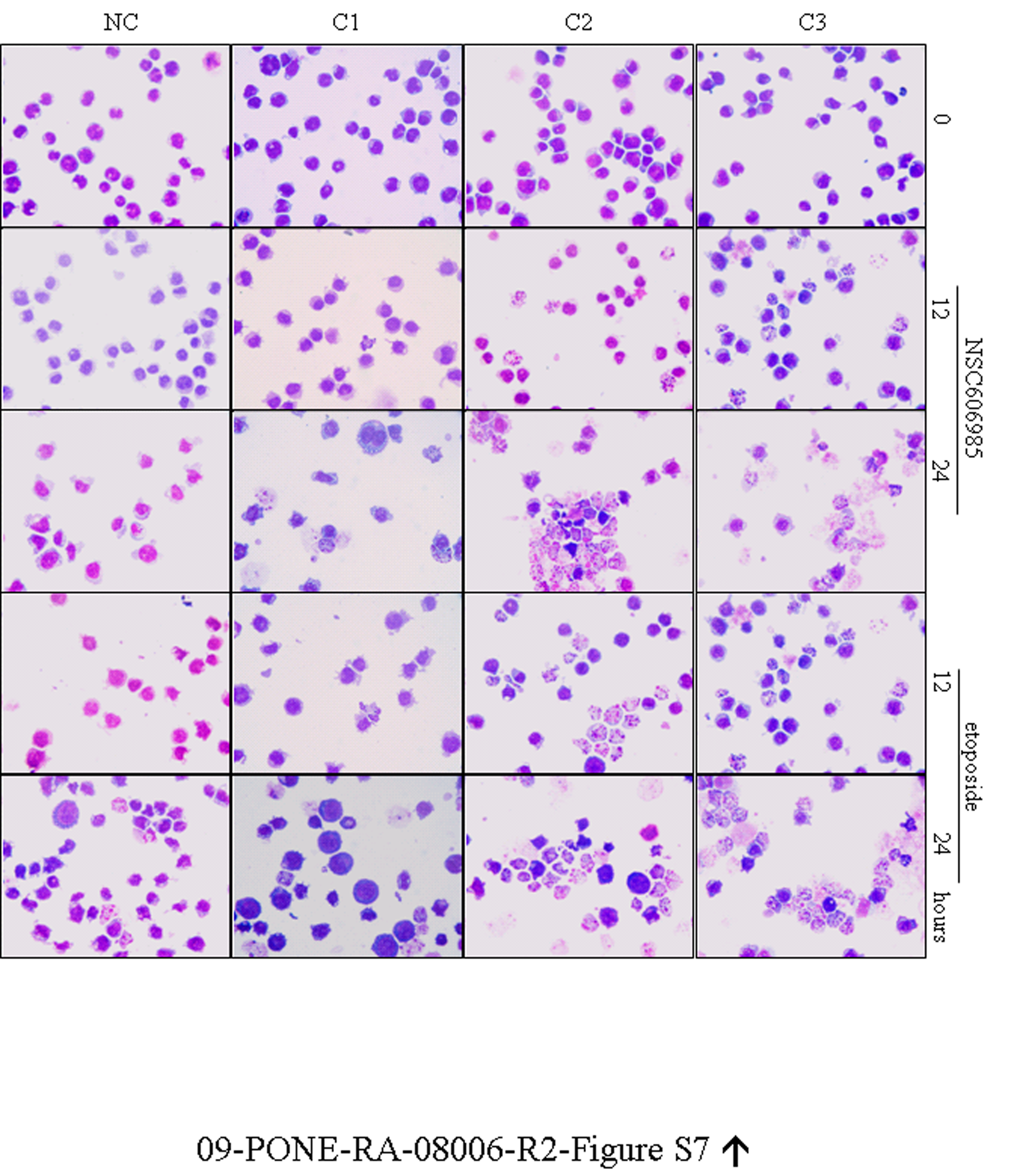

Supplement: Figure S7 — U937 cells with stable transfections of C1-C3 or NC were treated with 2 µM etoposide or 200 nM NSC606985 for hours as indicated, cell morphological features were examined under microscope after Wright's staining. (9.37 MB TIF) [file pone.0006552.s008.tif]

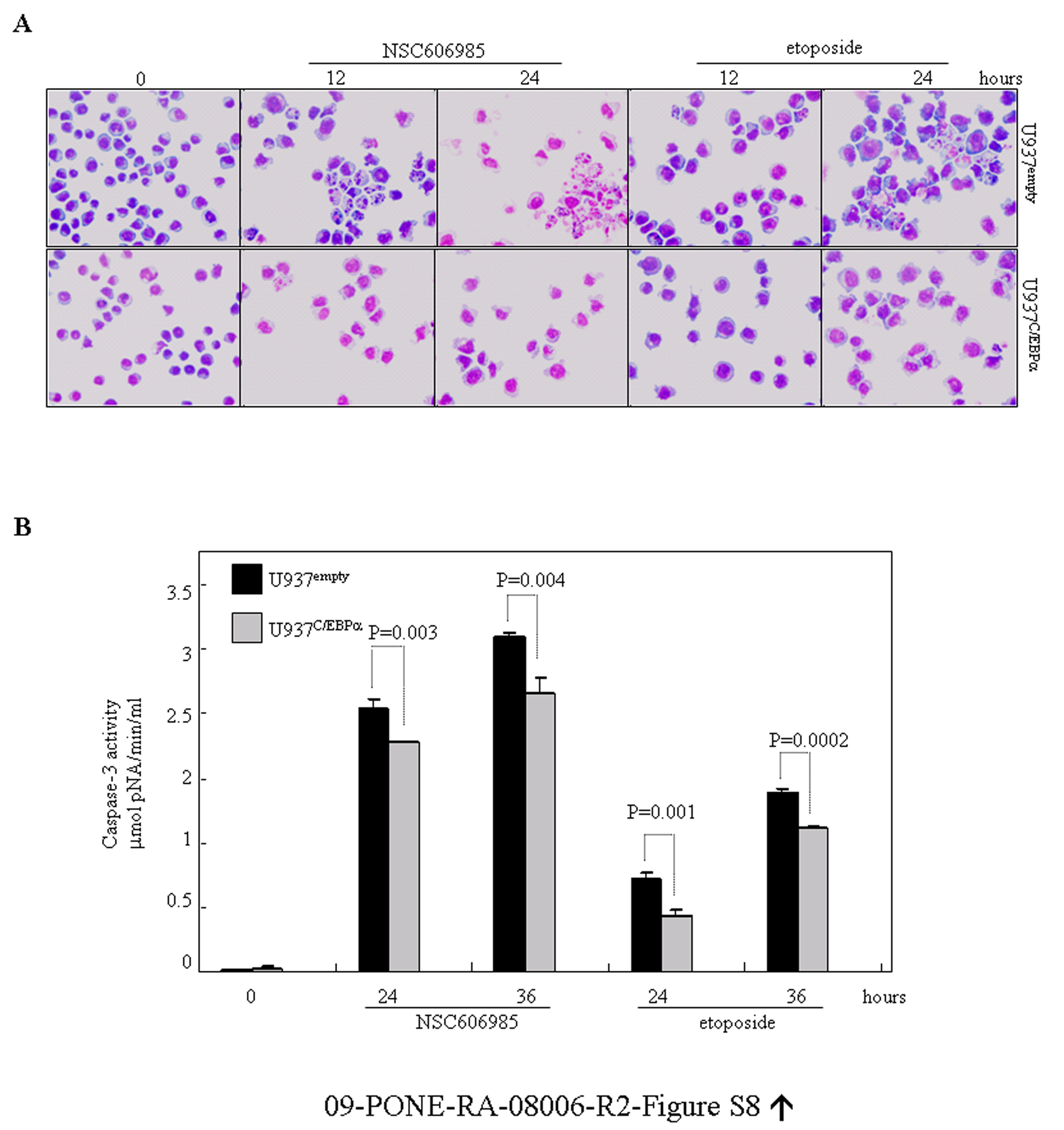

Supplement: Figure S8 — After pre-incubation in the absence of tetracycline for 8 days, U937empty and U937C/EBPα cells were treated with 200 nM NSC606985 or 2 µM etoposide for hours as indicated, cell morphological features were examined by microscope after Wright's staining of cells (A), and the caspase-3 activation was measured as described in materials and methods (B). The values represent mean±S.D. of triplicate in an independent experiment, which was repeated more than three times with the same results. (8.09 MB TIF) [file pone.0006552.s009.tif]
